# Social and Reproductive Behavior of Captive Malayan Tapirs’ (*Tapirus indicus*): Interactions with Maternal Experience and Environmental Conditions

**DOI:** 10.1038/s41598-020-60429-0

**Published:** 2020-03-05

**Authors:** Kalai Arasi Arumugam, Marina Mohd. Top, Wan Norhamidah Wan Ibrahim, Christina D. Buesching, Geetha Annavi

**Affiliations:** 1 Biology Department, Faculty of Science, Universiti Putra Malaysia, 43400 Serdang, Selangor Darul Ehsan Malaysia; 2 Wildlife Conservation Research Unit, Department of Zoology, Recanati-Kaplan Centre, University of Oxford, Tubney House, Abingdon Road, Tubney, Abingdon, Oxfordshire OX13 5QL UK

**Keywords:** Behavioural ecology, Animal behaviour

## Abstract

Malayan tapirs are listed as endangered and are bred in captivity under governmental management. The success of captive breeding programs varies and the underlying causes are unclear. Here, we investigate how tapir reproduction is affected by previous breeding experience, enclosure type/size and visitor numbers so that appropriate steps can be taken to achieve self-sustaining captive populations. Data on social and reproductive behaviors were collected from six tapirs (three males, three females), from different breeding centers in Peninsular Malaysia for 18 weeks. Results revealed that social and reproductive behavior of both sexes was significantly influenced by social and environmental conditions. Larger enclosure size tended to increase social and reproductive behaviors, whereas high number of visitors reduced initial interaction between males and females. No specific breeding month was confirmed; however, reproductive behaviors were highest in April. Overall, this study contributes to a better understanding of the relationships between social and reproductive behaviors, and captive environments on Malayan tapirs. In future, frequency of sexual interactions should be monitored regularly to identify animals exhibiting below-average frequency and who might, therefore, be prone to reproductive difficulties.

## Introduction

Despite several long-term studies of Malayan tapir (*Tapirus indicus*) in captivity^1–3^ and in the wild^4^, there is little research on the reproductive behavior of this species that investigate what factors affect the breeding success of this species. Malayan tapirs are threatened and endangered throughout their geographic regions due to habitat destruction, illegal logging and hunting pressure. In addition, their low reproductive rate has perpetuated the decline of Malayan tapir populations^5^. Therefore, a better understanding of their reproductive behavior will not only aid in conservation measures, but will also greatly improve our knowledge of their ecology and evolution.

Although previous studies have yielded important new insights into the reproductive biology^6^, anatomy and systematics^7^, as well as the activity pattern^8,9^ of Malayan tapir, no detailed studies exist on the reproductive behavior of this species. Particularly, studies investigating how environmental factors and maternal experience affect social behaviors and pair bonds are lacking. Currently, it is not known whether Malayan tapirs have a specific reproductive season, how their future breeding success is affected by maternal experience, and what male and female behaviors are associated with reproduction. Most mammal species have a discrete breeding season^10–13^, when particularly male reproductive behaviors can be observed more frequently^13,14^. For example, male *Giraffa Camelopardalis* initiate and perform sexual advancement more frequently in the presence of non-pregnant females compared to pregnant ones^14^. In captive environments^15,16^, especially when these places are open to visiting members of the public^17,18^, the stress caused by visitors who engage in harassing, teasing the animals or calling out in a loud voice^8,19^ tend to suppress reproductive activity, thus resulting in serious problems in captive breeding and species conservation management programs^20^. Anthropogenic noise is particularly hazardous and can have a significant impact on the physiological well-being and reproductive success of many endangered animals in captivity such as in captive koalas, *Phascolarctos cinereus*^21^, Indian Blackbuck, *Antelope cervicapra*^22^, giant pandas, *Ailuropoda melanoleuca*^23,24^ *inter alia*. However, stress in captivity is not caused by visitors alone but can also be due to housing or other environmental factors and can arise also by social affiliation^8,19^. For instance, in species in the same biological order as tapirs such as captive black rhinocereos (*Diceros bicornis*), smaller enclosure sizes are associated with reduced breeding success in males due to an increase in dominance- associated traits^17^. Meanwhile the increase in stress-related behaviors such as chasing, stereotypic behaviors and mouthing in female captive black rhinoceros could be due to the percentage of wall surrounding their enclosure^17^. The high exposure to zoo visitors within their enclosure causes fear^17^ and the increase in fighting between breeding partners results in correspondingly elevated mean corticoid levels^25^. Nevertheless, in some other species such as chimpanzees exposure to zoo visitors was found to have a neutral or even positive and enriching effect as human visitors represented the possibility to obtain food rather being a direct stressor^19^. In Malayan tapirs, however, enclosure characteristics have been reported to significantly affect their general behaviors and activity budget^8^, but the effects (including visitor-related disturbance levels) on tapir social and reproductive behaviors are currently not quantified.

Here, we report the social and reproductive behaviors of captive male and female Malayan tapirs, focusing on how these behaviors are affected by captive environment (enclosure type, enclosure size, temperature and number of visitors), month and maternal experience (parity and pregnancy).

## Methodology

### Study sites and subject

This study was performed at Sungai Dusun Wildlife Reserve (SDWR; GPS reference: 3° 40′ 45.3″ N, 101° 23′ 49.2504″ E) and two local zoos: Zoo Negara (ZN; 3° 12′ 25.5996″ N, 101° 45′ 24.2244″ E), and Zoo Melaka (ZM; 2° 16′ 35.5332″ N, 102° 17′ 56.04″ E) in Peninsular Malaysia (see Table 1 for site details) over a period of 18 weeks between both, the dry and the rainy season from 7^th^ March and 4^th^Aug 2016, alternating between two-week observation periods at each location to achieve equal and temporally-comparable coverage because the weather in Peninsular Malaysia is quite changeable. Three pairs of adult Malayan tapirs from three different sites were observed for a total of 6 weeks per pair (see Table 2 for details of study animals). During this study, the female tapirs from SDWR and ZN were confirmed to be pregnant during examination by in-house veterinary doctors, whereas the other female from ZM was confirmed not to be pregnant. Enclosure types were categorized as semi-natural and artificial enclosures. Semi-natural enclosures were surrounded by forest with no visitors permitted (and thus the amount of human-generated noise was minimal) while artificial enclosures were surrounded by buildings and traffic, and were open to visitors, resulting in substantial human-originating noise pollution (as confirmed by the first author during her direct observation sessions of the animals as well as later in the lab during videotape analysis that also recorded sound). Although we are aware that tapirs rely heavily on olfactory cues and signals in spatial orientation, predator avoidance and intra-specific communication^26^ and are also affected by vibrations caused by generators, water filters, construction noise, concerts, etc^27^, it was unfortunately not possible for us to quantify these potential further sources of visitor-related disturbance in this present study. This is because we did not have access to the necessary specialized equipment to measure vibrations (e.g. with a geophone), olfactory cues in the air (e.g. with thermal desorption tubes), or exact determinant of noise levels (which would require very sensitive and standardized sound recorders and acoustic analysis equipment). Detailed descriptions of the layout of each enclosure are available in Arumugam *et al*. (2018).

**Table 1:** Details of Malayan tapir enclosures included in the study.

Place	Surroundings	Outdoor Enclosure Size	Outdoor Substrate	Indoor Enclosure Size	Indoor Substrate	Feeding and resting sites	Visual barrier	Visitor access
SDWR	forest	728 m^2^	concrete floor, grass and soil	16 m^2^	concrete floor	indoor	high	no
ZN	buildings and traffic	765 m^2^	grass and soil	none	—	outdoor	high	yes
ZM	buildings and traffics	1189 m^2^	grass and soil	^*^15 m^2^	concreate floor	outdoor	low	yes

^*^Indoor in ZM was used only to keep the pair separately during zoos non-operating hour.

**Table 2:** Details of tapirs included in this study.

Subject	Sex	Age	Birth Place	Status of female	Parity of female	Number of offspring with same partner
Pair A	M	9	Captive	Pregnant	Parous	3
	F	13	Wild			
Pair B	M	7	Captive	Non-pregnant	Nulliparous	None
	F	12	Wild			
Pair C	M	9	Captive	Pregnant	Parous	3
	F	12	Wild			

Sex M = Male, F = Female, Parous = female had history given birth to offspring, Nulliparous = female had no history of given birth to offspring.

### Ethical statement

All animal handling procedures were approved by the University of Putra Malaysia ethics committee (Reference: UPM/IACUC/AUP-R033/2016). This study was completed in strict accordance with relevant guidelines and regulations from the Department of Wildlife and National Parks (PERHILITAN), Zoo Negara and Zoo Melaka. Experimental protocols and applications for behavioral study were approved by the Department of Wildlife and National Parks (PERHILITAN), Zoo Negara and Zoo Melaka Committees.

### Behavioral observation

An ethogram based on tapir literature^28^ and other Perissodactyla^9,29–32^ was constructed by incorporating some modifications after an initial behavioral observation period in November 2015 at ZN prior to actual data collection (Table 3), during which the observer also got acquainted with various type of tapirs’ behavior and could pre-test and optimise the behavioral recording methodology. Behaviors were recorded continuously for 8 consecutive hours from 0900 to 1700 (i.e., during zoos operating hours) from each pairs using digital video cameras (Brand: Sony, Model: FDR-AXP35) positioned on the visitor observation decks at ZN and ZM, and several motion-detector activated camera traps in video mode (Brand: Scout Camera, Model: DTC-560 K) mounted on trees and steel bars in both, the outdoor and the indoor enclosure at SDWR. Camera traps were set to their maximum recording length of one minute after each trigger event with minimum trigger intervals of 1 s. All recorded data were then transferred onto datasheet and analysed individually using scan sampling in 30 second intervals^33^.

**Table 3:** Ethogram of male and female Malayan tapir social and reproductive behavior.

Behavioral Grouping		Behavior Subgrouping	Description
Social Behavior (common for male and female)	Initiation	Touching	Movement of the proboscis onto conspecific body part.
		Follow	The animal travels the same direction behind the conspecific.
		Approach	Forward movement toward conspecific in a straight or curving path.
		Licking	Movement of tongue on conspecific body part.
		Rubbing	Moving body back and forth on conspecific body part.
	Antagonistic	Aggression	The animal showing violent movement either by biting the flesh, rapid kicking or pushing away the conspecific.
		Moving away	The animal travels in a direction away from a conspecific.
	Vocalization	Vocal	Sound produced through the oral or sinus cavity during approaching or moving away from conspecific.
Reproductive Behaviors (Male Specific)	Identification	Smelling of female’s urine or dung	Smelling of urine or dung; may be followed by flehmen response.
		Smelling of vagina	Male smells female vaginal area; followed by licking and perform flehmen.
	Courtship	Chin Resting	Male rests his head on the rump or back of the female.
		Touch Feet	Movement of male’s proboscis to touch female’s hindquarters.
		Erection	Protrusion of the erected penis from the prepuce.
	Copulation	Mounting	Male straddles female’s back with forelegs while standing on hindlegs while leaning his breast on the female’s quarters.
		Mating	The female lowered her back quarters and allow male to begin thrusting.
Reproductive Behavior (Female specific)	*Identification	Smelling of male’s urine or dung	Smelling of urine or dung of male tapir followed by flehmen response.
		Smelling of anus or penis area	Female smells male tapir’s rectal or penis area; followed by licking and flehmen
	*Courtship	Chin Resting	Female rests her head on the rump or back of the male
		Squirting Urine	Female contracts the vagina, squirting urine on the male’s face
	*Copulation	Mounting	Female straddles male’s back with forelegs while standing on hindlegs while leaning its chest on the male’s quarters

*male-like sexual behavior patterns shown in female.

Outdoor weather variables (temperature and humidity) were measured using HygroThermometer Clocks (Extech Instruments, Model: 445702), and the number of visitors in front of the viewing points of tapirs’ enclosure were counted using click counters every 35 minutes^8^. Noise level was categorized by ear (always by the same person to minimize inter-observer variation) as low, medium or high.

### Data analysis

The frequency of each behavior was summed for each observational day per 30 s observational period for the duration of the respective behavior and the number of visitors, temperature and humidity variables were standardized to a mean of zero^34^. The weather variables were inter-correlated (i.e. if temperature (°C) increased, humidity (%) decreased; r = −0.77, p < 0.05) and generally yielded similar results. Thus, we used only temperature for our main analyses (but see Table S1 for humidity). All statistical analyses were run in R Statistical Package Version 3.3.2. We fitted a multiple linear regression model using the lm() function to analyze social and reproductive behavior with the fixed effects and model averaging based on information criteria, AICc^35^ in MuMIn package^36^. Female maternal experience (reproductive status: pregnant and non-pregnant; parity: parous and nulliparous), four environmental factors (temperature, enclosure size, enclosure type and number of visitors) and month were included as fixed effects. The frequency of social (contact between male and female; initiation, antagonistic, vocalization) and reproductive behavior of male (identification and courtship) and female (combining identification, courtship and copulation due to less clear behavioral distinctions between them with fluent transitions) specific groupings were modeled as the response variable on the Y-axis (Table 3). Male specific copulation behavior was excluded in the statistical analysis as too few were observed during this study. We used an information-theoretic (IT) approach to select the most plausible model and to estimate the importance of each fixed effect^37^. The models were ranked by AICc value as top model if ranked ∆AICc ≤ 7^37^. We then calculated the relative Akaike weight (ω) (exp [−0.5*∆AICc]), divided by the sum of the likelihoods for all models considered^35^. The 95% confident intervals for model-averaged parameter estimates were calculated using the model.avg function in R. The relative importance of each fixed effect was calculated as the total ω of all plausible models. Significant fixed effects were selected if the confident interval did not overlap zero.

## Results

### Month

**Figure 1:**
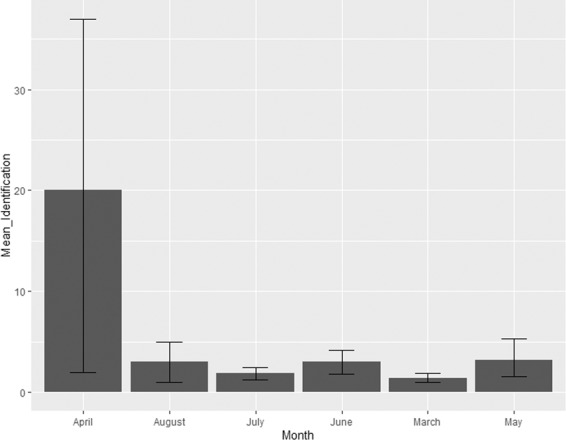
Mean frequency of male identification behaviors grouped into month. Month April had the highest frequency of identification behavior compared to other months.

None of the social behaviors varied significantly with month, indicating a non-seasonal pattern of social engagement by tapirs in captivity. Only one reproductive behavior was influenced by month (Table 4). Identification behavior peaked during the month of April and was low in all the other tested months (Fig. 1).

**Table 4:** Model-averaged parameter estimates over all submodels with Delta Akaike’s Information Criterion (ΔAICc) <7 testing the relationship between variables and grouped social and reproductive behaviors for male and female Malayan tapir. ß (CI) = Estimated value (95% Confidence Interval) and RI = Relative Importance. Bold estimates have a confidence interval that does not overlap with zero. Fixed effects: Status (Pregnant = 1; Non-Pregnant = 0), Parity (Pair A = 1; Pair B = 0; Pair C = 1), Month (March = 3; April= 4; May= 5; June= 6; July= 7; August= 8), Enclosure Size (SDWR (728 m^2^) & ZN (765 m^2^) = 1; ZM (1189 m^2^) = 2), Enclosure Type (Semi-natural = 0; Artificial = 1).

Explanatory variables	A. Initiation Behavior		B. Antagonist Behavior		C. Vocalization Behavior		D. Male –Identification Behavior		E. Male – Courtship Behavior		F. Female Reproductive Behavior	
	ß (CI)	RI	ß (CI)	RI	ß (CI)	RI	ß (CI)	RI	ß (CI)	RI	ß (CI)	RI
Intercept	−1.16 (−7.32, 4.99)	—	1.14 (−0.66, 2.92)	—	14.8 (4.34, 25.4)	—	0.35 (−3.12, 3.87)	—	0.12 (−2.22, 2.43)	—	0.87 (−1.85, 3.61)	—
Temperature	0.22 (−0.37, 0.83)	0.32	0.15 (−0.20, 0.50)	0.34	−0.45 (−2.54, 1.64)	0.28	−0.02 (−0.39, 0.36)	0.26	0.07 (−0.21, 0.33)	0.28	−0.03 (−0.18, 0.12)	0.26
Visitor	**−0**.**81** **(−1**.**39**, **−0**.**24)**	**0**.**95**	−0.26 (−0.60, 0.07)	0.52	−0.28 (−2.32, 1.72)	0.27	−0.12 (−0.48, 0.26)	0.30	0.02 (−0.25, 0.28)	0.26	−0.07 (−0.22, 0.07)	0.36
Enclosure Size	**2**.**86** **(0**.**41**, **5**.**33)**	**0**.**63**	0.57 (−0.74, 1.94)	0.43	2.56 (−5.78, 10.9)	0.43	**1**.**49** **(0**.**09**, **2**.**92)**	**0**.**64**	**1**.**08** **(0**.**29**, **1**.**86)**	**0**.**65**	0.31 (−1.44, 2.06)	0.49
Enclosure Type	**6**.**30** **(4**.**99**, **7**.**63)**	**1**.**00**	−0.58 (−1.35, 0.19)	0.52	**−8**.**23** **(−12**.**9**, **−3**.**60)**	**1**.**00**	0.57 (−0.46, 1.58)	0.41	0.28 (−0.38, 0.94)	0.33	−0.09 (−0.50, 0.31)	0.28
Month	−0.34 (−0.80, 0.11)	0.52	−0.087 (−0.37, 0.18)	0.33	−0.99 (−2.51, 0.53)	0.47	**−0**.**26** **(−0**.**52**, **−0**.**00)**	**0**.**74**	−0.11 (−0.29, 0.06)	0.44	−0.12 (−0.24, 0.00)	0.78
Status	**2**.**70** **(0**.**54**, **4**.**84)**	**0**.**93**	0.74 (−0.43, 1.93)	0.48	**−5**.**95** **(−11**.**6**, **−0**.**34)**	**0**.**74**	0.79 (−0.88, 2.56)	0.40	−0.05 (−1.18, 1.08)	0.29	0.41 (−0.11, 0.94)	0.53
Parity	**−2**.**86** **(−5**.**32**, **−0**.**45)**	**0**.**32**	−0.57 (1.94, 0.73)	0.22	−2.57 (−10.9, 5.78)	0.22	**−1**.**49** **(−2**.**92**, **−0**.**09)**	**0**.**32**	**−1**.**08** **(−1**.**86**, **−0**.**29)**	**0**.**32**	−0.92 (−2.29, 0.45)	0.68

### Maternal experience

**Figure 2:**
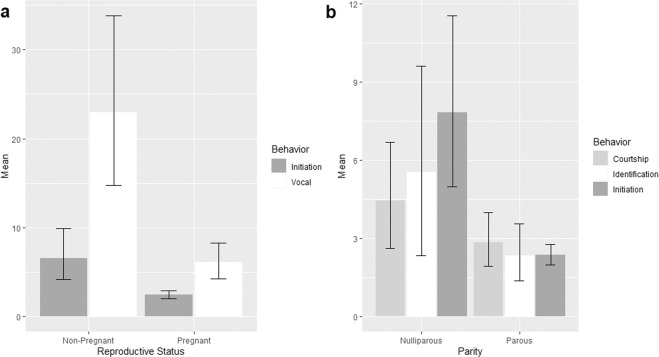
(**a**) Mean frequency of initiation and vocal behaviors influenced by female reproductive status of either pregnant or non-pregnant, both of the significant behaviors showed higher in frequency in non-pregnant female and its resident male. (**b**) mean frequency of male courtship and identification behaviors and initiation behavior were influenced by female reproductive history of its parity, nulliparous female and its resident male showed highest frequency of all the three significant behaviors.

The mean frequency between pairs for initiation and vocal behaviors were significantly affected by female reproductive status (pregnant versus non-pregnant; Table 4). The non-pregnant female and her resident male (pair B) exhibited a high frequency of initiation and vocal behaviors compared to the two pregnant females and her male partners (pairs A and C) (Fig. 2a). We found that the nulliparous female and her resident male (pair B) showed a higher frequency of initiation between each other compared to the parous females and their resident male (pairs A and C). Similarly, the mean frequency of identification and courtship behavior were higher in the male kept with a nulliparous female (pair B) compared to males kept with parous females (pairs A & C; Table 4; Fig. 2b).

### Environmental factor effects (enclosure type, enclosure size, temperature and visitor numbers)

**Figure 3:**
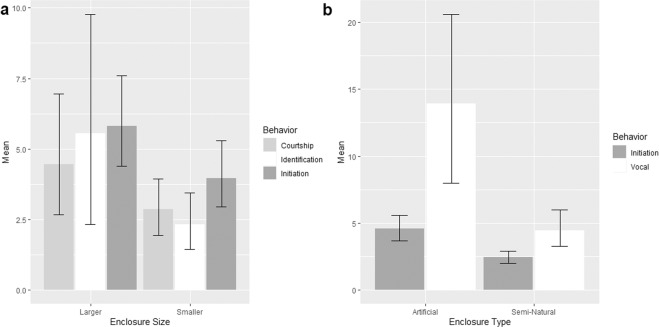
(**a**) Mean frequency of male courtship and identification behaviors and initiation behavior influenced by enclosure size (small = 728 m^2^ & 765 m^2^; large = 1189 m^2^), enclosure with largest space showed highest mean frequency, (**b**) mean frequency of initiation and vocal behaviors influenced by types of enclosure, the pairs showed more frequent of both significant behavior in artificial enclosure compared to semi-natural.

**Figure 4:**
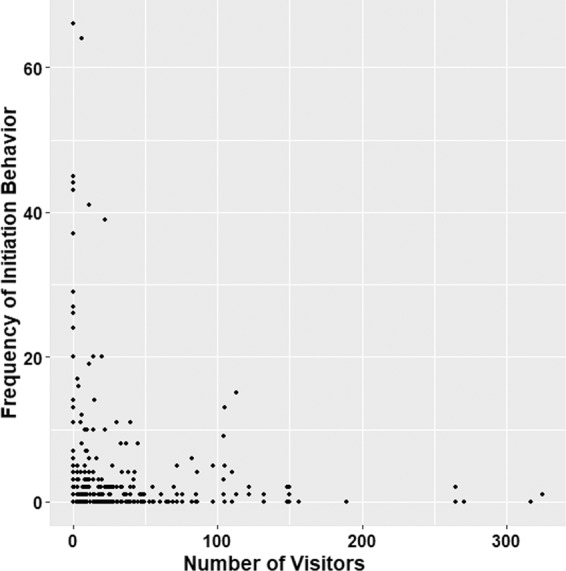
Frequency of initiation behavior influenced by number of visitors. Graph showing large frequency of initiation activity took place when number of visitor was between 0–50.

Enclosure size was correlated positively with identification and courtship behaviors in male tapirs (Fig. 3a) as well as the frequency of initiation behavior in males and females (Table 4). In contrast, an increase in visitor numbers caused a decrease in initiation behavior (Table 4; Fig. 4). Tapirs in artificial enclosures engaged more frequently in initiation and vocalization behaviors than tapirs in semi-natural enclosures (Table 4; Fig. 3b). Temperature did not affect social or reproductive behavior.

## Discussion

Initiation behaviors were seen as the first step to develop social bonding between tapirs. We noticed that males typically showed the first initiation behaviors such as touching, licking and rubbing when they encountered a female, and females mostly approached and touched the males, but less often followed, rubbed or licked. If the male would repeat the initiation behavior or not was largely dependent on how the female reciprocated (either through counter initiation or antagonistic behaviors). Identification and courtship behaviors were followed mostly after initiation behaviors. However, as we did not analyse behavioral sequences in this study, the detailed behavioral progression of pair-bonding in Malayan tapirs need to be investigated in more detail in future studies. Identification by smelling female’s urine or vaginal area followed by a flehmen response is a common way for a male mammal to determine female estrus^38^.

There were several factors, which possibly affected initiation, identification and courtship behaviors. Females’ maternal experience, followed by environmental factors, was the primary reason to induce the male-female sexual receptivity^39^ and increase the intensity of behavioral estrus signs^40^. Female reproductive status of being pregnant or non-pregnant influenced the initiation behavior between the paired tapirs, which is also very commonly seen in other terrestrial mammals^14,30,41,42^^,^. However, initiation behaviors were seen also between the partners in the pregnant pairs A and C, which likely indicates that this behavior can also function to reduce aggression between couples and/or strengthen pair-bonds as previously suggested to prevent injuries or fatality during their encounters^41,42^. Female B who did not have any maternal experience was more receptive during estrus than pregnant females A and C reflecting similar observations by Metrione, (2010) in Southern white rhinoceros (*Ceratotherium simum simum*). It was also proven in domestic goats *Capra hircus* males that engage in investigation behavior such as anogenital sniffing more often when exposed to zero maternal experienced females compared to parous or pregnant females^43^. Therefore, we suggest that the reason why Male B displayed higher frequency of identification and courtship behaviors throughout the study period may have been due to the presence of receptive females that act as androgen inducer^44^.

Enclosure size positively affected initiation behaviors, whereby the larger semi-natural enclosure enabled tapirs to engage in approaching and following behaviors as they would do in the wild^27^. Large enclosures also allowed the male and female to separate themselves more effectively during aggressive encounters associated with breeding^17^. Larger enclosures also allowed the tapirs in this study to perform courtship and pre-courtship behaviors effectively. Nevertheless, although tapirs in artificial enclosures were successfully engaged in initiation behavior showing even when in the presence of visitors, the frequencies of initiation behaviors were higher during periods of low visitor numbers (<150), and the associated reduction in visitor-generated noise levels (i.e., voices, footsteps, paper crinkling etc) surrounding the enclosure. In the presence of visitors and background noises, all tapirs were found to be continuously vigilant, either in a standing, sitting or a lying down position, without any social interaction with their partner. Unfortunately, due to logistical reasons, it was impossible for us to collect data at night during the predominant natural activity period of tapirs^45^. Nevertheless, considering the increasing popularity of extended visitor access to zoos^19^ and animal displays with reversed day/night light cycle^27^, the potentially problematic effects of visitor-related disturbance on the reproductive behavior of this species (mirroring observations in other species of similar conservation concern^21–24^ etc.) need to be highlighted. For animal welfare as well as captive breeding success, it is thus crucial to keep also nocturnal or crepuscular animals away from visitor disturbance, and thus to allow social behaviors to take place during any time of day. Although here we could only interpret on noise levels, nocturnal mammals such as tapirs typically rely heavily on olfactory signals in intra-specific communication^46^, and thus smell-related disturbance from visitors also needs to be investigated in future studies.

In addition during visiting time, it is also critical to ensure all tapirs, but particularly the females, to be kept at least one meter away from visitors as suggested in the Tapiridae Care Manual^27^ to avoid physical and mental stress resulting in miscarriages in pregnant females.

Antagonistic behaviors were found not to be influenced by any of the tested parameters. Any displayed antagonistic behaviors were likely due to attempts of individuals to increase levels of arousal of their opposite-sex partner^47^, or in the case of the pregnant females in pairs A and C - to avoid contact with their mates, and these behaviors are thus part of the normal intra-specific behavioral communication repertoire rather than a direct reaction to visitors and/or other environmental factors.

Although all reproductive behaviors were most frequently observed in male B during copulation events, only mounting was observed and no successful mating happened with female B. At each mating event, male B failed to show thrusting, which was likely due either to his immaturity and inexperience^32^, or his lack in morphological height, as male B was much smaller than female B and was therefore not able to reach the female B’s vaginal area to begin thrusting. From personal communication with the zoo keeper of pair B, we were informed that this pair did not produce any offspring in the time they have lived together. Based on that, if the body sizes of the paired male and female are not compatible, we believe that there will be no possibility for successful reproduction in tapirs. Consequently, this may lead to severe reproductive problems such as cystic endometrial hyperplasia, leiomyomas (of the cervix, uterus and ovary), adenoma, para-ovarian cysts and/or hydromucometra^48^ in tapirs, particularly in those females who do not reproduce for a long period of time, making future reproduction unlikely even if paired with a more suitable mate.

Apart from that, many mammals in the wild have a specific breeding season or month^10,12^ whereas others do not^49,50^. Previous studies have reported no evidence of seasonality in other tapir species^51,52^, but it is still unclear in Malayan tapirs. We observed all males performing identification behavior on their female partners during every month of the study period between March and August. However, identification behaviors were highest specifically during the month of April, likely indicating that April is the month when the female showed high stimulus value (female attractiveness) such as changes in genital morphology or changes in olfactory cues provided in the urine, feces, and vaginal discharge^53^.

Although we did not record vocal behavior systematically in the framework of this present study, female tapirs appeared to prefer vocal communication; while male tapirs were more interested in physical interaction. Animals emitting vocalizations can signal the physiological and psychological status of the caller^54^, and female tapirs were observed to vocalize regularly as a warning to threaten the male to stay away whereas males threatened females by using physical aggressiveness more often such as biting, kicking or pushing the female away and used vocalizations less often (unpubl. data). Males were usually heard vocalizing only during initiation behavior (following). A further study on types of vocalization associated with male and female behaviors however is highly necessary to obtain a better understanding of tapir vocal communication related to their social and reproductive needs in captivity.

## Conclusion

The results of this study contribute to the understanding of the relationships between reproduction and social behavior, and the influence of environmental factors on reproductive behavior in captivity. Our findings showed that, in captivity, the social and reproductive activity patterns depend mainly on the female’s maternal experience as well as environmental conditions. Being in a larger enclosure with fewer visitors and associated lower anthropogenic noise levels helped particularly non-pregnant and nulliparous females with the advancement of initiation behaviors or so-called consortship into breeding behaviors. The pregnant females and the resident male tended to alleviate aggression and dominance by initiating social interactions as a defensive strategy to protect themselves from injuries similar to mares^41^. Different types of vocal communication were noted throughout the study at different events during the social and reproductive encounters which need further investigation.

Identification behaviors were highest in April contributing evidence that female Malayan tapirs may have the highest sexual stimulus value in April. This potential seasonality in Malayan tapir reproductive biology needs to be investigated further in future studies. Despite showing a successful behavioral sequence up to the last stage (i.e., thrusting), one male could not achieve successful mating as it was much smaller than the resident female. Consequently, it could lead to female health problems, which are potentially hazardous if females are not mated for a long period of time, annihilating also future reproductive capacity. Therefore, introducing the male or female to a novel and physically compatible mating partner could help to increase the changes of mating activity resulting in a self-sustaining population. Future studies need to investigate in more detail the effects of visitor-induced (noise-) disturbance, particularly with a focus on the role of vocal communication in tapir reproduction.

## Supplementary information

Table S1.

## Data Availability

Data will be deposited in DRYAD before submission of a final version of the manuscript.
