# Supplementary material for: Social and Reproductive Behavior of Captive Malayan Tapirs’ (Tapirus indicus): Interactions with Maternal Experience and Environmental Conditions
Source: Sci Rep. 2020 Mar 5;10:4117. doi: 10.1038/s41598-020-60429-0 (PMC7058076; doi:10.1038/s41598-020-60429-0)
Supplement: Supplementary file 1 — Table S1. [file 41598_2020_60429_MOESM1_ESM.pdf]

Social and Reproductive Behavior of Captive Malayan Tapirs' (*Tapirus indicus*): Interactions with Maternal Experience and Environmental Conditions

Kalai Arasi Arumugam, Marina Mohd. Top @ Mohd. Tah., Wan Norhamidah Wan Ibrahim, Christina D. Buesching and Geetha Annavi

Supplementary Material

Table S1: Model-averaged parameter estimates over all submodels with Delta Akaike's Information Criterion (ΔAICc) < 7 testing the relationship between variables and grouped social and reproductive behaviors for male and female Malayan tapir. Result of humidity variable substituted with temperature variable.

**Explanatory variables**	**A. Initiation Behavior**	**B. Antagonist Behavior**	**C. Vocalization Behavior**	**D. Male – Identification Behavior**	**E. Male – Courtship Behavior**	**F. Female Reproductive Behavior**
	**ß (CI)**	**ß (CI)**	**ß (CI)**	**ß (CI)**	**ß (CI)**	**ß (CI)**
Intercept	-1.21 (-7.45, 5.05)	1.11 (-0.80, 2.99)	14.56 (4.02, 25.32)	0.33 (-3.18, 3.86)	0.12 (-2.21, 2.44)	0.84 (-1.85, 3.56)
Humidity	-0.23 (-0.84, 0.38)	-0.15 (-0.52, 0.20)	-0.32 (-2.43, 1.77)	-0.09 (-0.46, 0.29)	0.07 (-0.20, 0.34)	-0.04 (-0.20, 0.11)
Visitor	**-0.81 (-1.38, -0.24)**	-0.26 (-0.59, 0.08)	-0.32 (-2.35, 1.67)	-0.12 (-0.49, 0.26)	0.01 (-0.25, 0.28)	-0.08 (-0.22, 0.07)
Enclosure Size	**2.93 (0.45, 5.39)**	0.61 (-0.74, 2.02)	2.76 (-5.65, 11.01)	**1.51 (0.09, 2.94)**	**1.07 (0.28, 1.86)**	0.33 (-1.40, 2.06)
Enclosure Type	**6.30 (4.99, 7.63)**	-0.58 (-1.35, 0.19)	**-8.22 (-12.89, -3.60)**	0.56 (-0.46, 1.58)	0.29 (-0.38, 0.94)	-0.09 (-0.50, 0.31)
Month	-0.35 (-0.80, 0.11)	-0.087 (-0.37, 0.18)	-0.96 (-2.48, 0.56)	**-0.26 (-0.52, -0.0009)**	-0.11 (-0.29, 0.64)	-0.12 (-0.24, 0.005)
Status	**2.74 (0.57, 4.88)**	0.78 (-0.41, 1.99)	**-5.96 (-11.62, -0.34)**	0.80 (-0.84, 2.55)	-0.06 (-1.19, 1.07)	0.41 (-0.12, 0.95)
Parity	**-2.93 (-5.39, -0.45)**	-0.61(2.03, 0.74)	-2.76 (-11.06, 5.61)	**-1.51 (-2.94, -0.09)**	**-1.07 (-1.86, -0.29)**	-0.93 (-2.29, 0.43)
